# Prone Positioning During Venovenous Extracorporeal Membrane Oxygenation for Acute Respiratory Distress Syndrome With COVID-19: A Meta-Analysis

**DOI:** 10.7759/cureus.53049

**Published:** 2024-01-27

**Authors:** Minjin Shen, Jiaping Huai

**Affiliations:** 1 Department of Gastroenterology, Affiliated Jinhua Hospital, Zhejiang University School of Medicine, Jinhua, CHN; 2 Department of Critical Care Medicine, Affiliated Jinhua Hospital, Zhejiang University School of Medicine, Jinhua, CHN

**Keywords:** meta-analysis, ards, covid-19, v-v ecmo, prone positioning

## Abstract

The available literature has furnished substantial evidence indicating the favorable outcomes of prone positioning (PP) on oxygenation parameters among patients afflicted with coronavirus disease 2019 (COVID-19). However, there is a notable disparity in the reported influence of PP on the overall outcomes of COVID-19 patients undergoing venovenous extracorporeal membrane oxygenation (V-V ECMO) for acute respiratory distress syndrome (ARDS) across studies. This article has been prepared in adherence with Preferred Reporting Items for Systematic Review and Meta-Analyses (PRISMA) guidelines. MEDLINE, Embase, and Cochrane databases were utilized for data retrieval. The primary endpoint was to evaluate the cumulative survival rate among COVID-19 patients receiving V-V ECMO, comparing those who received PP to those who did not. Secondary endpoints included the duration of intensive care unit (ICU) stay, ECMO duration, and mechanical ventilation duration. A total of 15 studies involving 2286 patients were analyzed in the meta-analysis. PP significantly improved the cumulative survival rate (0.48, 95% CI: 0.40-0.55); risk ratio (RR) of 1.24 (95% CI: 1.11-1.38).PP during ECMO for COVID-19 patients yielded favorable outcomes in terms of 60-day survival, 90-day survival, ICU survival, and hospital survival. In contrast, patients who underwent PP had longer ECMO duration (8.1 days, 95% CI: 6.2-9.9, p<0.001) and mechanical ventilation duration (9.6 days, 95% CI: 8.0-11.2, p<0.001). PP demonstrated improved survival in COVID-19 patients with ARDS receiving V-V ECMO. However, additional well-designed prospective trials are warranted to further explore the effects of this combination on survival outcomes in COVID-19 patients.

## Introduction and background

In December 2019, a novel viral respiratory illness called coronavirus disease 2019 (COVID-19) emerged, triggering a worldwide pandemic. COVID-19 is a highly communicable respiratory disease primarily spread through contact, respiratory droplets, and potentially through airborne transmission in certain contexts, although the exact mechanisms are not yet fully elucidated [1]. A significant proportion of individuals infected with COVID-19 experience manageable respiratory symptoms and eventually achieve complete recovery [2]. However, approximately 18% of cases progress to severe or critical conditions [3], with a mortality rate ranging from 53% to 67% [4, 5].

Currently, the available therapeutic options for COVID-19 are limited, and in cases where conventional treatments, such as recruitment maneuvers and strategies, do not produce satisfactory results [6], the use of extracorporeal membrane oxygenation (ECMO) becomes essential for the provision of temporary organ support. Previous experiences with patients suffering from moderate-to-severe acute respiratory distress syndrome (ARDS) of various origins have shown that early implementation of prone positioning (PP) offers significant benefits, including improved oxygenation, better alignment of ventilation and blood flow, reduced risk of ventilator-induced lung injury, and enhanced survival rates [7,8]. In non-intensive care unit (ICU) settings, awake PP has proven safe and may lead to decreased respiratory rate and improved oxygenation, potentially delaying the need for intubation in COVID-19 patients [9]. PP has demonstrated promising advantages within the intensive care unit (ICU) for patients undergoing non-invasive ventilation or high-flow nasal cannula therapy, with or without the administration of sedation [10]. Although both venovenous extracorporeal membrane oxygenation (V-V ECMO) and PP have individually demonstrated positive outcomes for COVID-19 patients with ARDS, the combined safety and efficacy of V-V ECMO and PP remain unclear in the existing literature. Hence, we conducted a meta-analysis to evaluate the survival outcomes of COVID-19 patients treated with PP during ECMO, aiming to improve our comprehension of the treatment relationship and assess patient survival within this protocol.

## Review

Methods

Search Strategy and Study Identification

Following the recommended reporting standards of Preferred Reporting Items for Systematic Review and Meta-Analyses (PRISMA), we carried out a comprehensive meta-analysis [11]. We conducted a comprehensive search across the MEDLINE, Embase, and Cochrane databases, spanning from December 1, 2019, to August 1, 2023. We employed various keywords and their variations, including "extracorporeal membrane oxygenation (ECMO, extracorporeal life support, ECLS, membrane oxygenation, or extracorporeal oxygenation)," "prone position (positioning therapy)," and "COVID-19 (SARS-CoV-2, 2019 novel coronavirus disease)." The detailed search strategy can be found in Appendix 1. No language restrictions were set, enabling the inclusion of studies in different languages. Furthermore, we meticulously reviewed the bibliographies of potentially pertinent articles to identify any supplementary sources that could contribute to our research. The titles and abstracts of all identified results were independently assessed for eligibility by both authors in a rigorous and independent manner. In cases where an article was considered potentially suitable, both authors independently reviewed the full text for final inclusion. Any disagreements between the authors were resolved through consensus-based discussions.

Inclusion and Exclusion Criteria

This meta-analysis included original studies that fulfilled the subsequent eligibility criteria. Table 1 shows the inclusion and exclusion criteria.

**Table 1:** Inclusion and exclusion criteria in the meta-analysis V-V ECMO - venovenous extracorporeal membrane oxygenation

Inclusion criteria	Exclusion criteria
From December 1, 2019, to August 1, 2023	Duplicate publications or datasets
A minimum of 5 patients	Animal, laboratory experiments, individual case reports
Patients aged >18 years with confirmed COVID-19 who received V-V ECMO	Correspondence letters, editorial contributions, comments, conference abstracts, meta-analyses, and literature reviews
Studies that clearly reported clinical characteristics and mortality outcomes in patients	Studies published in languages other than English without a translated version
Studies with primary data	

Data Extraction and Quality Assessment

Data extraction from the articles included in the analysis involved capturing crucial details such as the first authors' names, year of publication, region, methodological design, number of subjects, participant demographic characteristics, clinical variables, duration of mechanical ventilation, duration of V-V ECMO, and survival outcomes. In case of the need for additional information, the researchers were contacted for supplementary results.

Both authors conducted a comprehensive quality assessment, considering both the specific study parameters and the measured outcomes. The Newcastle Ottawa Scale (NOS) was employed to evaluate the risk of bias in each eligible study, employing a three-category system [12]: patient selection, comparability of study arms, and outcome assessment. Each category has a maximum score of nine points. Studies scoring above five points were selected for subsequent analysis. The certainty of evidence for each outcome was assessed using the Grading of Recommendations, Assessments, Developments, and Evaluations (GRADE) approach [13]. Discrepancies were resolved through mutual agreement between the researchers.

Outcomes of Interest

The primary endpoint was cumulative survival. Secondary endpoints included differences in 28-day survival, 60-day survival, 90-day survival, ICU survival, hospital survival, duration of mechanical ventilation, and duration of ECMO.

Statistical Analysis

The STATA software (version 14.0; StataCorp LLC, College Station USA) was used to investigate both primary and secondary endpoints. Forest plot graphs were used to visually represent the main meta-analytical results for each variable. Calculation of the overall risk ratio (RR) and its 95% confidence interval (CI) was performed using a random-effects model [14]. I2 statistics were used to determine the amount of heterogeneity in the included studies, which classified heterogeneity as low (values less than 25%), moderate (values between 25% and 75%), or significant (values greater than 75%). Cochran Q statistics, along with its p-values, were also used to examine heterogeneity [15]. The evaluation of publication bias included the implementation of Egger's and Begg's tests alongside the examination of a visual funnel plot to identify any indications of asymmetry [16].

Results

Study Selection

**Figure 1:**
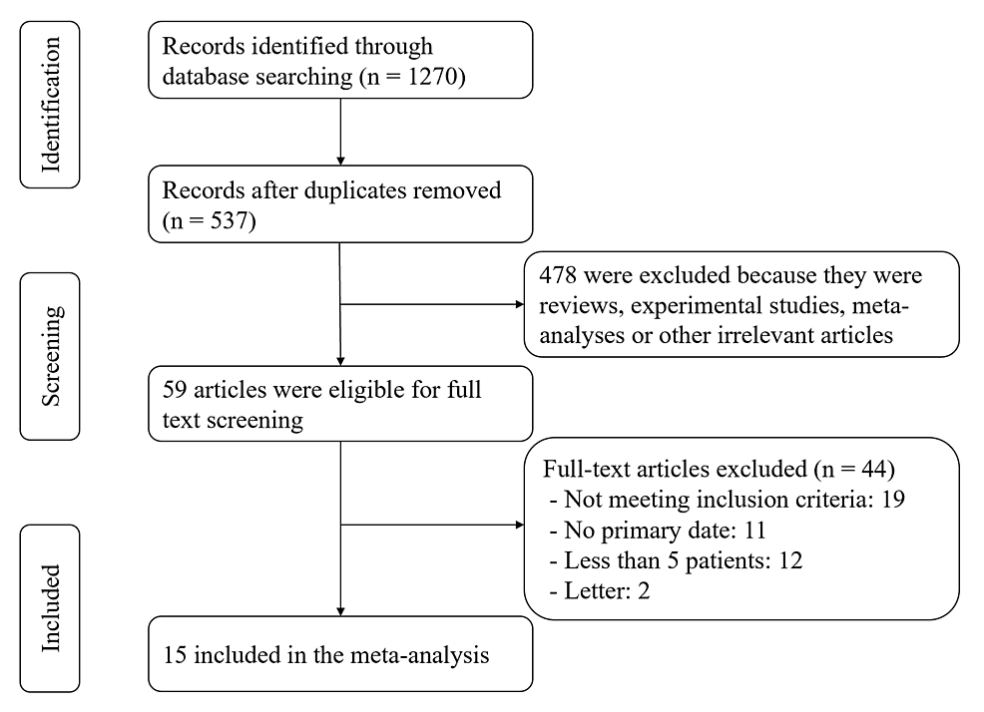
Selection process for the studies included in the meta-analysis

Upon executing the search strategy, 1270 articles were retrieved for screening based on titles and abstracts. Among them, 59 articles underwent full-text screening. Following the application of predefined inclusion and exclusion criteria, 15 studies [17-31] comprising a total of 2286 patients were included in this meta-analysis (Figure 1). In particular, five of them were observational studies with matched controls [17,20,27,29,30]. All of the studies selected for this meta-analysis were published from 2020 onwards.

Study Characteristics and Quality Assessment

Detailed descriptions of the 15 included studies are outlined in Table 2. Out of these studies, eleven were conducted in France, whereas the remaining four originated from Japan, China, and America. All of the studies were observational in design, with the majority being retrospective (13 retrospective, two prospective). The collective sample size consisted of 2286 COVID-19 patients who required ECMO, among whom 1487 underwent PP. The primary outcomes examined within the articles varied, with six focusing on hospital survival, three on 28-day survival, two on ICU survival, and two each on 60-day and 90-day survival. In terms of quality assessment using the NOS criteria (Table 3), all of the studies were considered to be of moderate to high quality.

**Table 2:** Characteristics of studies included in the meta-analysis * mean and standard deviation PP - prone positioning; ECMO - extracorporeal membrane oxygenation; SOFA - Sequential Organ Failure Assessment; BMI - body mass index; NA - not available

Study	Region	Study design	Overall, n	PP during ECMO, n	Age (years), median (IQR)	Male gender, n (%)	SOFA score, median (IQR)	BMI (kg/cm²), median (IQR)	PP before ECMO, n (%)		Duration of ECMO (days)	Primary outcome
Garcia et al. 2020 [17]	France	Retrospective	25	14	59 (49.5–63)	22 (88)	NA	32 (28.4-37.5)	25 (100)		10 (5–13)	28-day mortality
Jozwiak et al. 2020 [18]	France	Prospective	11	6	50 (38-59)	7 (64)	13 (9-14)	30 (26-32)	11 (100)		12 (8-25)	28-day mortality
Schmidt et al. 2020 [19]	France	Retrospective	83	67	49 (41–56)	61 (73)	12 (9–13)	30 (27.9-34.1)		NA	NA	60-day survival
Breton et al. 2020 [20]	France	Retrospective	13	7	50 (42-56)	10 (77)	9 (8-12)	NA	13 (100)		13 (8-18)	ICU mortality
Mori et al. 2020 [21]	Japan	Retrospective	5	5	54 (42-62)	4 (80)	NA	NA	5 (100)		10 (10-44)	ICU mortality
Nie et al. 2020 [22]	China	Retrospective	9	9	51.23 ± 9.72^*^	4 (44)	NA	NA	NA		NA	Hospital survival
Lebreton et al. 2021 [23]	France	Retrospective	302	193	52 (45-58)	235 (78)	12 (9-14)	30 (26.8–33.5)	285 (94)		14 (8-26)	90-day survival
Yang et al. 2021 [24]	China	Retrospective	73	51	63 (51-66)	46 (63)	NA	NA	NA		NA	Hospital mortality
COVID-ICU. 2021 [25]	France, Belgium, Switzerland	Prospective	269	184	NA	NA	NA	NA	NA		NA	90-day mortality
Nesseler et al. 2022 [26]	France	Retrospective	429	301	54 (46-60)	338 (79)	9 (8-12)	30 (27-34)	411 (96)		12 (8-21)	Hospital mortality
Zaaqoq et al. 2022 [27]	America	Retrospective	232	67	53 (43–60)	160 (69)	8 (5–10)	30 (27–36)	176 (76)		NA	Hospital survival
Hajage et al. 2022 [28]	France	Retrospective	269	184	54 (46–59)	207 (77)	8 (4–12)	30 (27–34)	240 (89)		11 (6–17)	60-day survival
Laghlam et al. 2022 [29]	France	Retrospective	24	10	52 (43–58)	18 (75)	10 (6–14)	30 (25–34)	24 (100)		14 (7–26)	28-day mortality
Massart et al. 2023 [30]	France	Retrospective	517	364	55 (47–61)	402 (78)	9 (7–12)	30 (27.5–34.3)	490 (95)		12.5 ± 12.76^*^	Hospital mortality
Textoris et al. 2023 [31]	France	Retrospective	25	25	55 (45–61)	18 (72)	7 (4–9)	30 (27.6–35.2)	25 (100)		23 (15–34)	Hospital mortality

**Table 3:** Quality assessment of the studies included (Ia) the exposed cohort was representative of the population; (Ib) the non-exposed cohort was drawn from the same population; (Ic) the exposure ascertainment was from secure records or a structured interview; (Id) no outcome events before the start of the study; (IIa) the cohorts were comparable for age and gender; (IIb) the cohorts were comparable for all additional factor(s) reported; (IIIa) cases were assessed from a secure record; (IIIb) follow-up was long enough; and (IIIc) follow-up was complete. NOS - Newcastle Ottawa Score

Study	Selection				Comparability		Assessment of outcome			NOS score
	Ia	Ib	Ic	Id	IIa	IIb	IIIa	IIIb	IIIc	
Garcia et al. 2020 [17]	*	*	*	*	*		*	*	*	8
Jozwiak et al. 2020 [18]	*	*	*	*			*	*	*	7
Schmidt et al. 2020 [19]	*	*	*	*			*	*	*	7
Breton et al. 2020 [20]	*	*	*	*			*	*	*	7
Mori et al. 2020 [21]	*		*	*			*	*	*	6
Nie et al. 2020 [22]	*		*	*			*	*	*	6
Lebreton et al. 2021 [23]	*	*	*	*			*	*	*	7
Yang et al. 2021 [24]	*	*	*	*			*	*	*	7
COVID-ICU. 2021 [25]	*	*	*	*			*	*	*	7
Nesseler et al. 2022 [26]	*	*	*	*			*	*	*	7
Zaaqoq et al. 2022 [27]	*	*	*	*			*	*	*	7
Hajage et al. 2022 [28]	*	*	*	*			*	*	*	7
Laghlam et al. 2022 [29]	*	*	*	*	*		*	*	*	8
Massart et al. 2023 [30]	*	*	*	*	*		*	*	*	8
Textoris et al. 2023 [31]	*	*	*	*			*	*	*	7

Primary Endpoint

**Figure 2:**
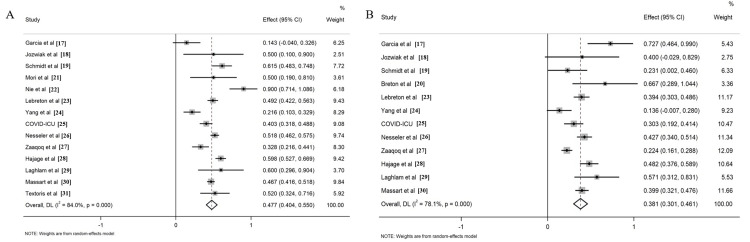
Forrest plots for the cumulative survival rate of COVID-19 patients with PP (A) and without PP (B) PP - prone positioning

**Figure 3:**
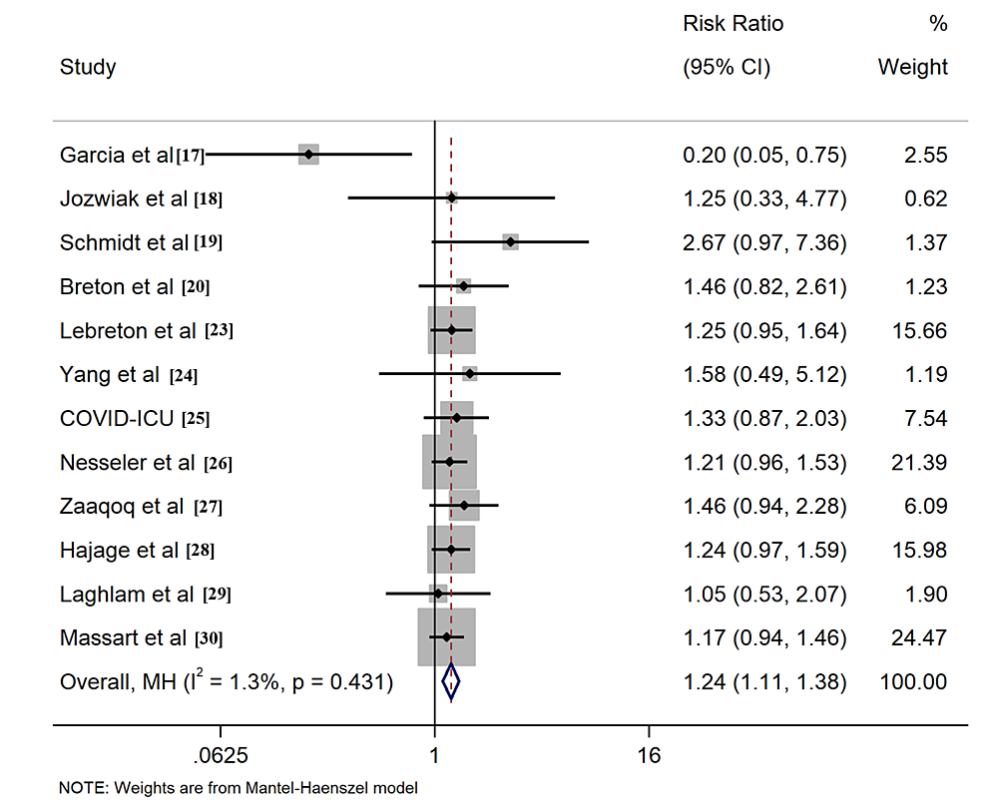
Forrest plots for comparison of cumulative survival in prone position and control group patients

The 15 included studies presented various survival data, encompassing various time points such as 28-day, 60-day, and 90-day survival, as well as ICU and hospital survival. The primary endpoint focused on pooled cumulative survival, reflecting the reported survival rate at the longest observed time point. For COVID-19 patients who underwent PP, the cumulative survival rate was 0.48 (95% CI: 0.40, 0.55, Figure 2A), while for those without PP, the survival rate was 0.38 (95% CI: 0.30, 0.46, Figure 2B). The synthesized data revealed a significantly higher cumulative survival rate in the PP group compared to the control group, with a risk ratio (RR) of 1.24 (95% CI: 1.11, 1.38, Figure 3).

Secondary Endpoints

**Figure 4:**
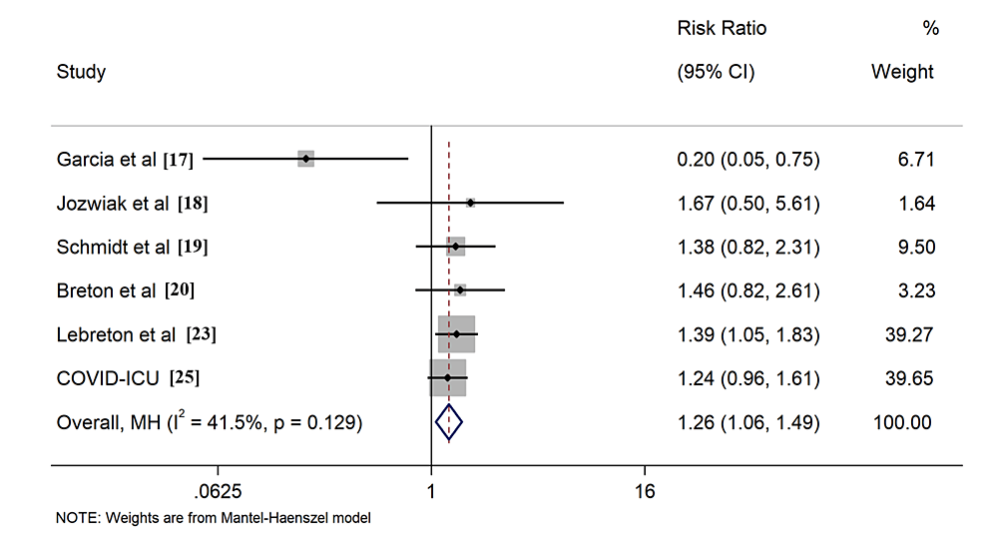
Forrest plots for comparison of ICU survival in prone position and control group patients

**Figure 5:**
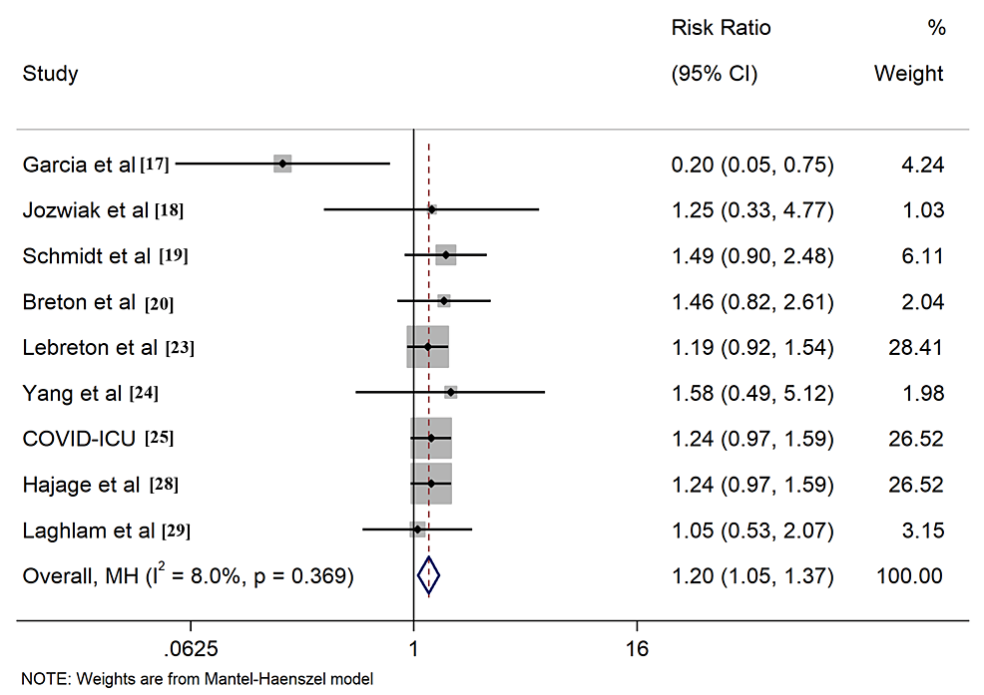
Forrest plots for comparison of 60-day survival in prone position and control group patients

**Figure 6:**
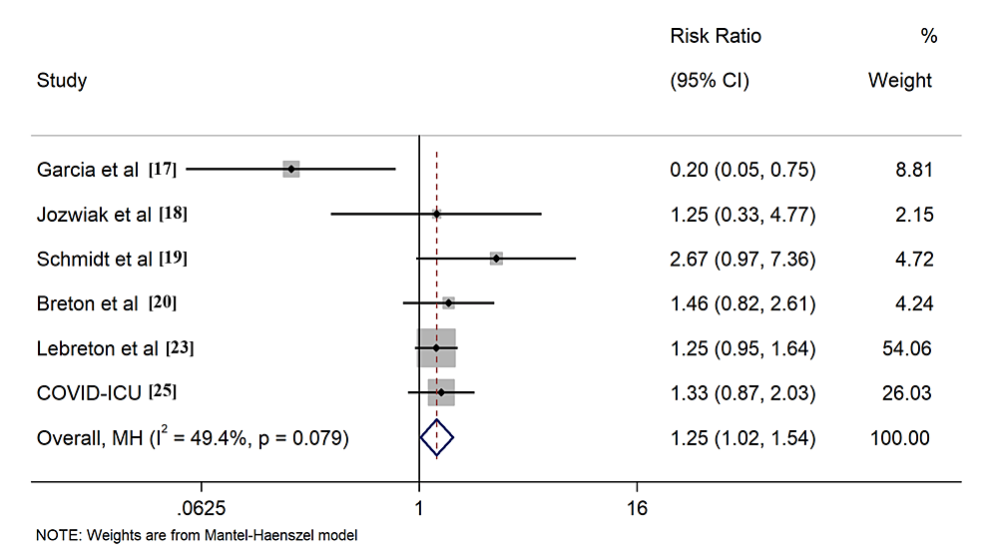
Forrest plots for comparison of 90-day survival in prone position and control group patients

**Figure 7:**
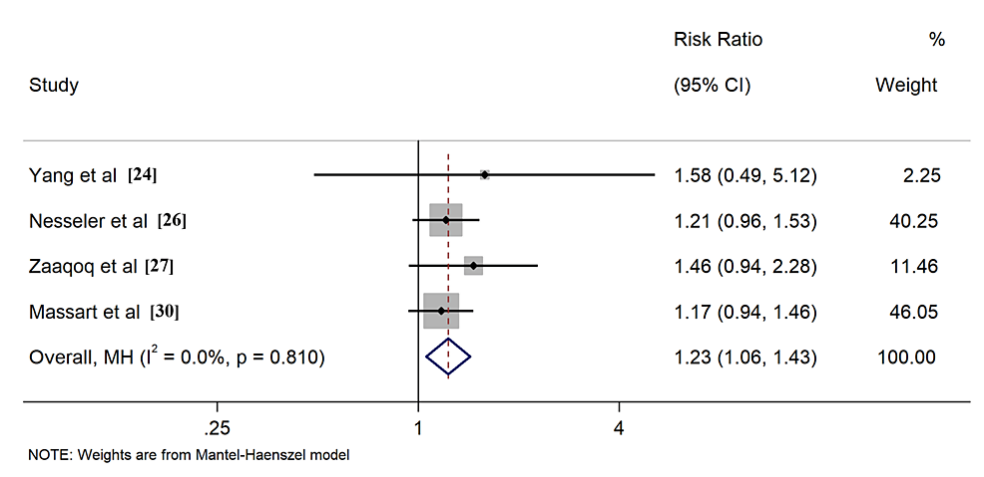
Forrest plots for comparison of hospital survival in prone position and control group patients

**Figure 8:**
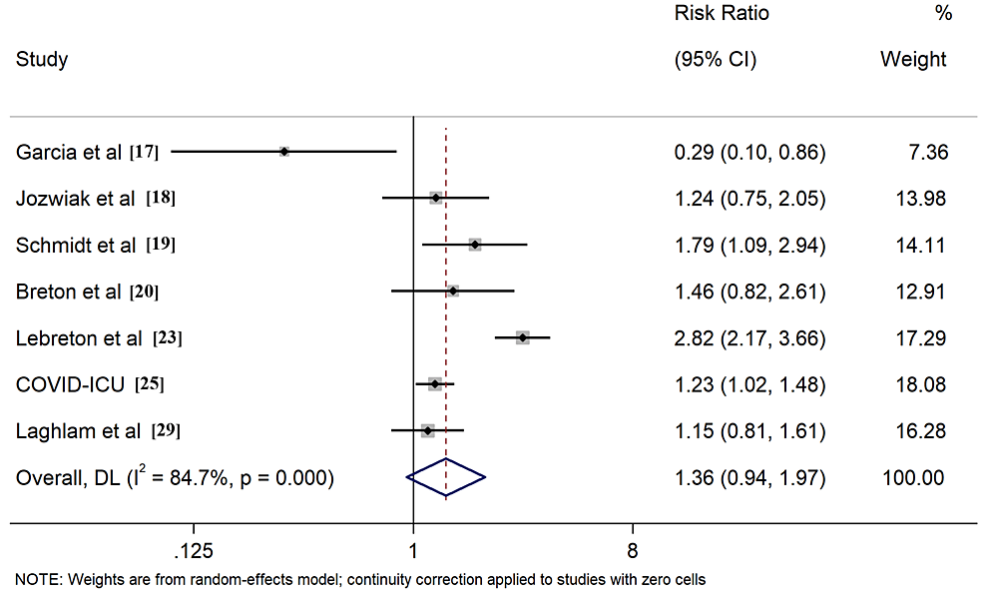
Forrest plots for comparison of 28-day survival in prone positioning and control group patients

**Figure 9:**
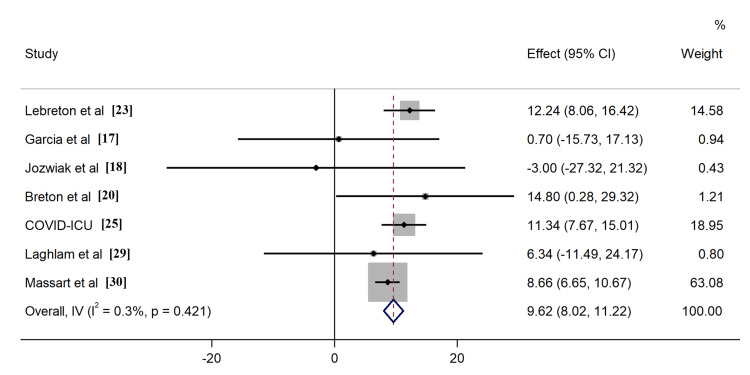
Forrest plots for the duration of mechanical ventilation

**Figure 10:**
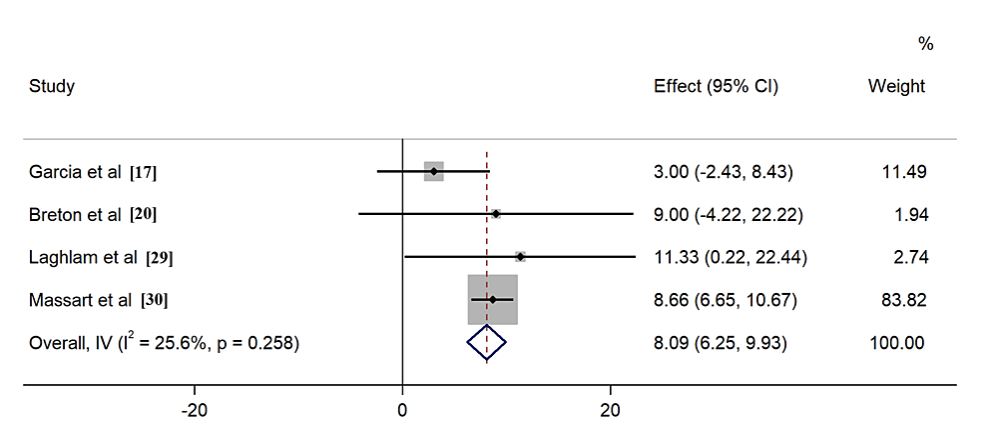
Forrest plots for the duration of ECMO ECMO - extracorporeal membrane oxygenation

All included studies reported the outcome of all-cause mortality, of which seven [17-20,23,25,29] described the 28-day survival, nine [17-20,23-25,28,29] provided 60-day survival, six [17-20,23,25] described 90-day survival, six [17-20,23,25] described ICU survival, and four [24,26,27,30] described hospital survival, respectively. Application of PP in ECMO COVID-19 patients resulted in improved ICU survival (RR: 1.26, 95% CI: 1.06-1.49, Figure 4). Comparable outcomes were found when evaluating survival rates at different time intervals, including 60-day, 90-day, and hospital survival (Figures 5-7). However, combined findings from seven studies did not demonstrate statistically significant results, suggesting a disparity in 28-day survival between PP and control groups (RR: 1.36, 95% CI: 0.94-1.97). Notably, there was significant heterogeneity, with an I2 of 84.7% (Figure 8). Conversely, the use of PP resulted in prolonged durations of mechanical ventilation and ECMO (mean difference 9.62 days [95% CI: 8.02-11.22] and 8.09 days [95% CI: 6.25-9.93], respectively; Figures 9-10).

Data from nine studies [17-21,25,26,30,31] were available regarding complications, including hemorrhage, thrombosis, infection, and mechanical complications. These complications were observed with comparable frequencies in both PP and control groups. Remarkably, no patients experienced any significant adverse events, such as accidental decannulation or displacement of the endotracheal tube. Due to variations in reporting methods among the studies, a comprehensive summary of adverse events could not be provided.

Publication Bias

**Figure 11:**
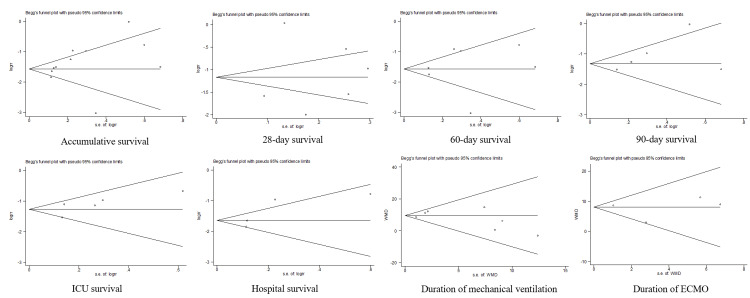
Begg's funnel plots

The funnel plots corresponding to the different outcomes displayed a globally balanced distribution; nevertheless, given the small number of studies, the existence of publication bias cannot be entirely excluded (Figure 11). The results of both Egger's and Begg's tests consistently yielded p-values of >0.05, indicating that the observed asymmetry lacks statistical significance.

Discussion

This meta-analysis, based on the evaluation of fifteen studies involving a total of 2286 patients, revealed a significant statistical discrepancy in cumulative survival rates between COVID-19 patients supported by V-V ECMO. The observed difference was attributed to the choice between PP and supine mechanical ventilation. With the exception of the 28-day survival rate, all other time intervals (including 60-day survival, 90-day survival, ICU survival, and hospital survival) consistently demonstrated the advantageous effects of prone positioning for COVID-19 patients undergoing V-V ECMO.

COVID-19 resulted in significant morbidity and mortality worldwide. Among COVID-19 patients who were hospitalized, 26% required admission to ICUs, with 88% of them needing mechanical ventilation to manage ARDS [32]. Recommendations published by Extracorporeal Life Support Organization (ELSO) suggest that patients experiencing severe or critical respiratory failure, unresponsive to conventional therapies, may necessitate the implementation of V-V ECMO support [33]. In the midst of the ongoing pandemic, the widespread implementation of PP has been observed in patients admitted with COVID-19 pneumonia and ARDS. This approach is rooted in the existing body of evidence demonstrating the mortality-reducing effects of PP in intubated individuals with moderate-to-severe ARDS not related to COVID-19 [34]. Existing research has indicated that PP yields favorable outcomes in COVID-19 patients, leading to an enhanced ratio of partial pressure of oxygen to fraction of inspired oxygen (PaO₂/FiO₂) and improved oxygen saturation (SpO₂) levels when compared to the supine position [35]. The adoption of PP during V-V ECMO treatment is both feasible and safe, offering potential benefits in enhancing oxygenation for individuals with ARDS. This approach achieves these positive outcomes by facilitating alveolar recruitment and mitigating hypoxemia-related complications associated with mechanical ventilation [36]. Nevertheless, it is important to weigh the potential benefits of prone positioning in V-V ECMO patients against the inherent risks of complications, particularly in centers lacking experience in its implementation.

In our analysis, we observed a higher survival rate (48%) compared to another study (31%) that did not utilize PP in COVID-19 patients undergoing V-V ECMO treatment [37]. Additionally, we found that COVID-19 patients who did not receive PP during V-V ECMO had a lower survival rate of 38% (95% CI: 30.1-46.1). The statistical analysis demonstrated a significant difference in the chances of survival between the PP and control groups. In addition, we also found that the patients who underwent PP can improve ICU survival, as well as hospital survival, and 60- and 90-day survival, except 28-day survival. No significant difference in 28-day survival was found in this meta-analysis, which might be attributed to the short follow-up time. However, both groups of patients differed in demographics, comorbidities, severity of COVID-19, and treatment provided, leading to possible bias in results. Moreover, a significant proportion of the studies included in our analysis did not provide comprehensive information regarding the duration and frequency of PP. We have also noticed some factors that contributed to the high heterogeneity, including factors with moderate or high certainty of association with increased mortality included patient factors, such as older age, male sex, and chronic lung disease; pre-cannulation disease factors, such as longer duration of symptoms, longer duration of invasive mechanical ventilation, higher PaCO_2_, and higher driving pressure; and center factors, such as less previous experience with ECMO. Consequently, it remains unclear whether the observed variability in this regard can be attributed to institutional workflow differences, as our review did not yield conclusive findings on this aspect. Given the absence of well-designed prospective studies investigating the effectiveness of PP during ECMO, it proves challenging to definitively exhibit the meaningful advantages associated with the integration of PP and ECMO.

The meta-analytical results revealed a significant prolongation in the duration of V-V ECMO support and mechanical ventilation among COVID-19 patients who underwent PP during V-V ECMO. It is probable that these patients had a higher severity of illness, which could explain the imperative for intervention and the extended periods of ECMO support and mechanical ventilation. Although several reports incorporated in this meta-analysis suggested a potential enhancement in PaO_2_/FiO_2_ as a result of prone positioning (PP), it is crucial to cautiously interpret these findings and consider them in conjunction with the broader analysis [17,29,30]. However, according to a previous study, PP with severe COVID-19-related ARDS resulted in a favorable oxygenation response in only 29% of cases [38]. On the flip side, it was observed that the beneficial effects on oxygenation during PP did not persist once patients returned to the supine position. Within six hours following re-supination, as highlighted in another study, the PaO_2_/FiO_2_ ratio resumed baseline levels [39]. This phenomenon could be attributed to the re-collapse of previously opened dorsal lung units during PP, leading to ventilation/perfusion mismatch and subsequent rebound hypoxemia. It is worth noting that the duration and frequency of PP varied among the included studies, as determined by the treating physicians, thus introducing potential bias into our findings.

Several limitations should be considered in this study. Firstly, all the findings were derived from observational studies, which may introduce a degree of uncertainty to our conclusions. Conducting a meta-analysis of observational studies presents inherent challenges due to variations in subjects, outcome definitions, study designs, and other factors, as well as potential data incompleteness and bias. A second limitation of this study stems from the scarcity of studies reporting precise information on the duration and session of PP. Moreover, the documentation of these details might have been subject to imprecision, as it relied on bedside clinical physicians without a standardized system and with uncertain accuracy. As a result, we were unable to examine the impact of PP duration on patient outcomes. Thirdly, we were unable to examine the occurrence of complications specifically associated with ECMO. Moreover, the survival analyses conducted to assess mortality were constrained by a short-term follow-up period and variations in the duration of death observations, which may have resulted in an underestimated prevalence of mortality and complications.

## Conclusions

In conclusion, this meta-analysis incorporated the largest cohort of COVID-19 patients who underwent PP during ECMO, providing valuable insights into the outcomes. The cumulative survival rate of 48% indicates that there may be significant clinical benefits for COVID-19 patients who receive PP in conjunction with ECMO. Nevertheless, given the considerable heterogeneity and the scarcity of evidence, it is vital to conduct additional well-designed studies in order to bolster the confidence and reliability of the conclusions.

## Appendices

**Table 4:** Search strategy for the meta-analysis

Search strategy
"Extracorporeal Membrane Oxygenation"[Mesh]
“Extracorporeal Membrane Oxygenation” [Title/Abstract] OR "ECMO "[Title/Abstract] OR "Extracorporeal Life Support"[Title/Abstract] OR “ECLS” [Title/Abstract] OR "membrane oxygenation"[Title/Abstract] OR “extracorporeal oxygenation” [Title/Abstract]
#1 OR #2 (“Extracorporeal Membrane Oxygenation” [Title/Abstract] OR "ECMO "[Title/Abstract] OR "Extracorporeal Life Support"[Title/Abstract] OR “ECLS” [Title/Abstract] OR "membrane oxygenation"[Title/Abstract] OR “extracorporeal oxygenation” [Title/Abstract]) OR ("Extracorporeal Membrane Oxygenation"[Mesh])
"Prone Position"[Mesh]
“prone position” [Title/Abstract] OR “positioning therapy” [Title/Abstract]
“Prone position” [Mesh] OR “prone position” [Title/Abstract] OR “positioning therapy” [Title/Abstract]
#3 and #6 ("Prone position"[Mesh Terms] OR "prone position"[Title/Abstract] OR " positioning therapy" [Title/Abstract]) AND (“Extracorporeal Membrane Oxygenation” [Title/Abstract] OR "ECMO "[Title/Abstract] OR "Extracorporeal Life Support"[Title/Abstract] OR “ECLS” [Title/Abstract] OR "membrane oxygenation"[Title/Abstract] OR “extracorporeal oxygenation” [Title/Abstract]) OR "Extracorporeal Membrane Oxygenation"[Mesh Terms])
"COVID-19"[Mesh]
“COVID-19” [Title/Abstract] OR "SARS-CoV-2 "[Title/Abstract] OR “2019 novel coronavirus disease” [Title/Abstract] OR "2019-nCoV"[Title/Abstract]
#3, #7 and #9 ("Prone position"[Mesh Terms] OR "prone position"[Title/Abstract] OR " positioning therapy" [Title/Abstract]) AND (“Extracorporeal Membrane Oxygenation” [Title/Abstract] OR "ECMO "[Title/Abstract] OR "Extracorporeal Life Support"[Title/Abstract] OR “ECLS” [Title/Abstract] OR "membrane oxygenation"[Title/Abstract] OR “extracorporeal oxygenation” [Title/Abstract]) OR "Extracorporeal Membrane Oxygenation"[Mesh Terms]) AND (“COVID-19” [Title/Abstract] OR " SARS-CoV-2 "[Title/Abstract] OR “2019 novel coronavirus disease” [Title/Abstract] OR "2019-nCoV"[Title/Abstract])

## References

[1] Wax RS, Christian MD (2020). Practical recommendations for critical care and anesthesiology teams caring for novel coronavirus (2019-nCoV) patients. Can J Anaesth.

[2] Struyf T, Deeks JJ, Dinnes J (2020). Signs and symptoms to determine if a patient presenting in primary care or hospital outpatient settings has COVID-19 disease. Cochrane Database Syst Rev.

[3] Sun P, Qie S, Liu Z, Ren J, Li K, Xi J (2020). Clinical characteristics of hospitalized patients with SARS-CoV-2 infection: a single arm meta-analysis. J Med Virol.

[4] Xu J, Yang X, Yang L (2020). Clinical course and predictors of 60-day mortality in 239 critically ill patients with COVID-19: a multicenter retrospective study from Wuhan, China. Crit Care.

[5] Xie J, Wu W, Li S (2020). Clinical characteristics and outcomes of critically ill patients with novel coronavirus infectious disease (COVID-19) in China: a retrospective multicenter study. Intensive Care Med.

[6] Fan E, Del Sorbo L, Goligher EC (2017). An official American Thoracic Society/European Society of Intensive Care Medicine/Society of Critical Care Medicine Clinical Practice Guideline: mechanical ventilation in adult patients with acute respiratory distress syndrome. Am J Respir Crit Care Med.

[7] Gattinoni L, Taccone P, Carlesso E, Marini JJ (2013). Prone position in acute respiratory distress syndrome. Rationale, indications, and limits. Am J Respir Crit Care Med.

[8] Gibson K, Dufault M, Bergeron K (2015). Prone positioning in acute respiratory distress syndrome. Nurs Stand.

[9] Carsetti A, Damia Paciarini A, Marini B, Pantanetti S, Adrario E, Donati A (2020). Prolonged prone position ventilation for SARS-CoV-2 patients is feasible and effective. Crit Care.

[10] Ding L, Wang L, Ma W, He H (2020). Efficacy and safety of early prone positioning combined with HFNC or NIV in moderate to severe ARDS: a multi-center prospective cohort study. Crit Care.

[11] Shamseer L, Moher D, Clarke M (2015). Preferred reporting items for systematic review and meta-analysis protocols (PRISMA-P) 2015: elaboration and explanation. BMJ.

[12] Stang A (2010). Critical evaluation of the Newcastle-Ottawa scale for the assessment of the quality of nonrandomized studies in meta-analyses. Eur J Epidemiol.

[13] Higgins JP, Green S (2008). Cochrane Handbook for Systematic Reviews of Interventions.

[14] Sun J, Freeman BD, Natanson C (2012). Meta-analysis of Clinical Trials. Principles and Practice of Clinical Research.

[15] Higgins JP, Thompson SG, Deeks JJ, Altman DG (2003). Measuring inconsistency in meta-analyses. BMJ.

[16] Egger M, Davey Smith G, Schneider M, Minder C (1997). Bias in meta-analysis detected by a simple, graphical test. BMJ.

[17] Garcia B, Cousin N, Bourel C, Jourdain M, Poissy J, Duburcq T (2020). Prone positioning under VV-ECMO in SARS-CoV-2-induced acute respiratory distress syndrome. Crit Care.

[18] Jozwiak M, Chiche JD, Charpentier J (2020). Use of venovenous extracorporeal membrane oxygenation in critically-ill patients with COVID-19. Front Med.

[19] Schmidt M, Hajage D, Lebreton G (2020). Extracorporeal membrane oxygenation for severe acute respiratory distress syndrome associated with COVID-19: a retrospective cohort study. Lancet Respir Med.

[20] Le Breton C, Besset S, Freita-Ramos S (2020). Extracorporeal membrane oxygenation for refractory COVID-19 acute respiratory distress syndrome. J Crit Care.

[21] Mori S, Ai T, Otomo Y (2020). Characteristics, laboratories, and prognosis of severe COVID-19 in the Tokyo metropolitan area: a retrospective case series. PLoS One.

[22] Nie Q, Ye A, Wei S (2020). Nursing management of severe COVID-19 patients undergoing extracorporeal membrane oxygenation combined with prone position ventilation. Heart Surg Forum.

[23] Lebreton G, Schmidt M, Ponnaiah M (2021). Extracorporeal membrane oxygenation network organisation and clinical outcomes during the COVID-19 pandemic in Greater Paris, France: a multicentre cohort study. Lancet Respir Med.

[24] Yang X, Hu M, Yu Y (2020). Extracorporeal membrane oxygenation for SARS-CoV-2 acute respiratory distress syndrome: a retrospective study from Hubei, China. Front Med.

[25] COVID-ICU Group (2021). Clinical characteristics and day-90 outcomes of 4244 critically ill adults with COVID-19: a prospective cohort study. Intensive Care Med.

[26] Nesseler N, Fadel G, Mansour A (2022). Extracorporeal membrane oxygenation for respiratory failure related to COVID-19: a nationwide cohort study. Anesthesiology.

[27] Zaaqoq AM, Barnett AG, Griffee MJ (2022). Beneficial effect of prone positioning during venovenous extracorporeal membrane oxygenation for coronavirus disease 2019. Crit Care Med.

[28] Hajage D, Combes A, Guervilly C (2022). Extracorporeal membrane oxygenation for severe acute respiratory distress syndrome associated with COVID-19: an emulated target trial analysis. Am J Respir Crit Care Med.

[29] Laghlam D, Charpentier J, Hamou ZA (2021). Effects of prone positioning on respiratory mechanics and oxygenation in critically ill patients with COVID-19 requiring venovenous extracorporeal membrane oxygenation. Front Med.

[30] Massart N, Guervilly C, Mansour A (2023). Impact of prone position in COVID-19 patients on extracorporeal membrane oxygenation. Crit Care Med.

[31] Textoris L, Gragueb-Chatti I, Daviet F (2023). Response to prone position in COVID-19 and non-COVID-19 patients with severe ARDS supported by vvECMO. J Clin Med.

[32] Wang D, Hu B, Hu C (2020). Clinical characteristics of 138 hospitalized patients with 2019 novel coronavirus-infected pneumonia in Wuhan, China. JAMA.

[33] Badulak J, Antonini MV, Stead CM (2021). Extracorporeal membrane oxygenation for COVID-19: updated 2021 guidelines from the Extracorporeal Life Support Organization. ASAIO J.

[34] Gill R, Wong T, Johnston LCA (2013). Prone positioning in severe acute respiratory distress syndrome (ARDS). J Intensive Care Soc.

[35] Chua EX, Zahir SM, Ng KT, Teoh WY, Hasan MS, Ruslan SR, Abosamak MF (2021). Effect of prone versus supine position in COVID-19 patients: a systematic review and meta-analysis. J Clin Anesth.

[36] Guérin C, Albert RK, Beitler J (2020). Prone position in ARDS patients: why, when, how and for whom. Intensive Care Med.

[37] Zhang Y, Wang L, Fang ZX, Chen J, Zheng JL, Yao M, Chen WY (2022). Mortality in patients with COVID-19 requiring extracorporeal membrane oxygenation: a meta-analysis. World J Clin Cases.

[38] Patel BV, Haar S, Handslip R (2021). Natural history, trajectory, and management of mechanically ventilated COVID-19 patients in the United Kingdom. Intensive Care Med.

[39] Gattinoni L, Tognoni G, Pesenti A (2001). Effect of prone positioning on the survival of patients with acute respiratory failure. N Engl J Med.

